# Clinical Presentations of Granulomatous Skin Reactions Following Tattooing and Cosmetic Micropigmentation: A Systematic Review

**DOI:** 10.7759/cureus.102227

**Published:** 2026-01-24

**Authors:** Yadavalli RD Rajan, Sesha Deepthi, Bhuvana Dosakayala, Sivani Mummadireddy, Sai Deekshitha Erri, Rahithya Alaparthi

**Affiliations:** 1 Plastic and Reconstructive Surgery, Sri Venkateswara Institute of Medical Sciences, Tirupati, IND; 2 Pathology, Konaseema Institute of Medical Sciences and Research Foundation, Amalapuram, IND; 3 Internal Medicine, Siddhartha Medical College, Vijayawada, IND; 4 Internal Medicine, RVM Institute of Medical Sciences and Research Centre, Siddipet, IND; 5 Internal Medicine, Sri Venkateswara Institute of Medical Sciences, Tirupati, IND

**Keywords:** cutaneous granuloma, micropigmentation, systemic sarcoidosis, tattoo ink, tattoo reaction

## Abstract

Tattooing and cosmetic micropigmentation are widely practiced procedures that are usually considered safe, but they can cause delayed granulomatous reactions and can comprise more than localized foreign-body reactions. This review synthesizes available evidence on granulomatous cutaneous reactions following tattooing and cosmetic micropigmentation, focusing on clinical presentation, histopathology, systemic associations, treatments, and outcomes. Across the included studies, granulomatous reactions occurred commonly with black and red pigments and presented as papular or nodular lesions with variable latency. Histological findings were consistent with non-caseating granulomas, and a substantial proportion of patients demonstrated ocular or systemic involvement, particularly sarcoidosis. Therapeutic approaches varied, most commonly involving immunosuppressive treatments, with generally favorable outcomes reported. Overall, granulomatous reactions associated with tattooing and micropigmentation, although uncommon, carry important clinical implications and should prompt consideration of systemic evaluation rather than being regarded as isolated cutaneous events.

## Introduction and background

Tattooing and cosmetic micropigmentation have become increasingly common worldwide, with a substantial proportion of the adult population now exposed to permanent intradermal pigments for decorative, cosmetic, or reconstructive purposes. Tattoos are widely regarded as safe procedures; however, a spectrum of cutaneous complications has been described, ranging from transient inflammatory reactions and infections to delayed hypersensitivity responses and granulomatous inflammation [1-3]. As the prevalence of tattooing continues to rise, so too does clinical recognition of uncommon but potentially significant adverse reactions.

Granulomatous reactions represent a distinct subset of tattoo-related complications and may manifest as localized foreign-body granulomas or as sarcoid-like reactions confined to tattooed skin. Clinically, these reactions often present as papules, nodules, plaques, or indurated lesions within the tattooed area, frequently with a delayed onset occurring months to years after pigment implantation [4,5]. Histopathologically, these lesions are characterized by non-caseating epithelioid granulomas, with or without visible pigment particles, making differentiation from cutaneous sarcoidosis and other granulomatous dermatoses challenging [6]. Of particular clinical importance is the recognized association between tattoo-associated granulomatous reactions and systemic sarcoidosis. Several reports have documented cases in which granulomatous tattoo reactions preceded, coincided with, or signaled the diagnosis of systemic disease, including pulmonary and ocular involvement [7,8]. This association raises important questions regarding whether tattoo pigments act as chronic immune triggers, unmask latent sarcoidosis, or represent a localized manifestation of a systemic granulomatous process. Ocular involvement, especially uveitis, has been repeatedly reported in association with tattoo granulomas, further underscoring the systemic implications of what may initially appear to be a cutaneous finding [9].

Despite growing recognition, the literature on granulomatous reactions following tattooing and cosmetic micropigmentation remains fragmented, consisting largely of case reports and small series with variable reporting of clinical features, histopathology, treatment approaches, and outcomes. The relative contributions of pigment color, composition, anatomical site, latency period, and host susceptibility remain incompletely understood, and no consensus exists on optimal diagnostic evaluation or management strategies. Additionally, cosmetic micropigmentation, although increasingly popular, has been comparatively underrepresented in published analyses. Given these uncertainties, a comprehensive synthesis of the existing evidence is warranted. This systematic review aims to collate and critically appraise published studies describing granulomatous cutaneous reactions following tattooing or cosmetic micropigmentation, with a focus on clinical presentation, histopathological patterns, systemic associations, treatment modalities, and outcomes. By integrating available data, this review seeks to enhance clinical awareness, support timely diagnosis, and identify gaps to guide future research in this evolving field.

## Review

**Figure 1:**
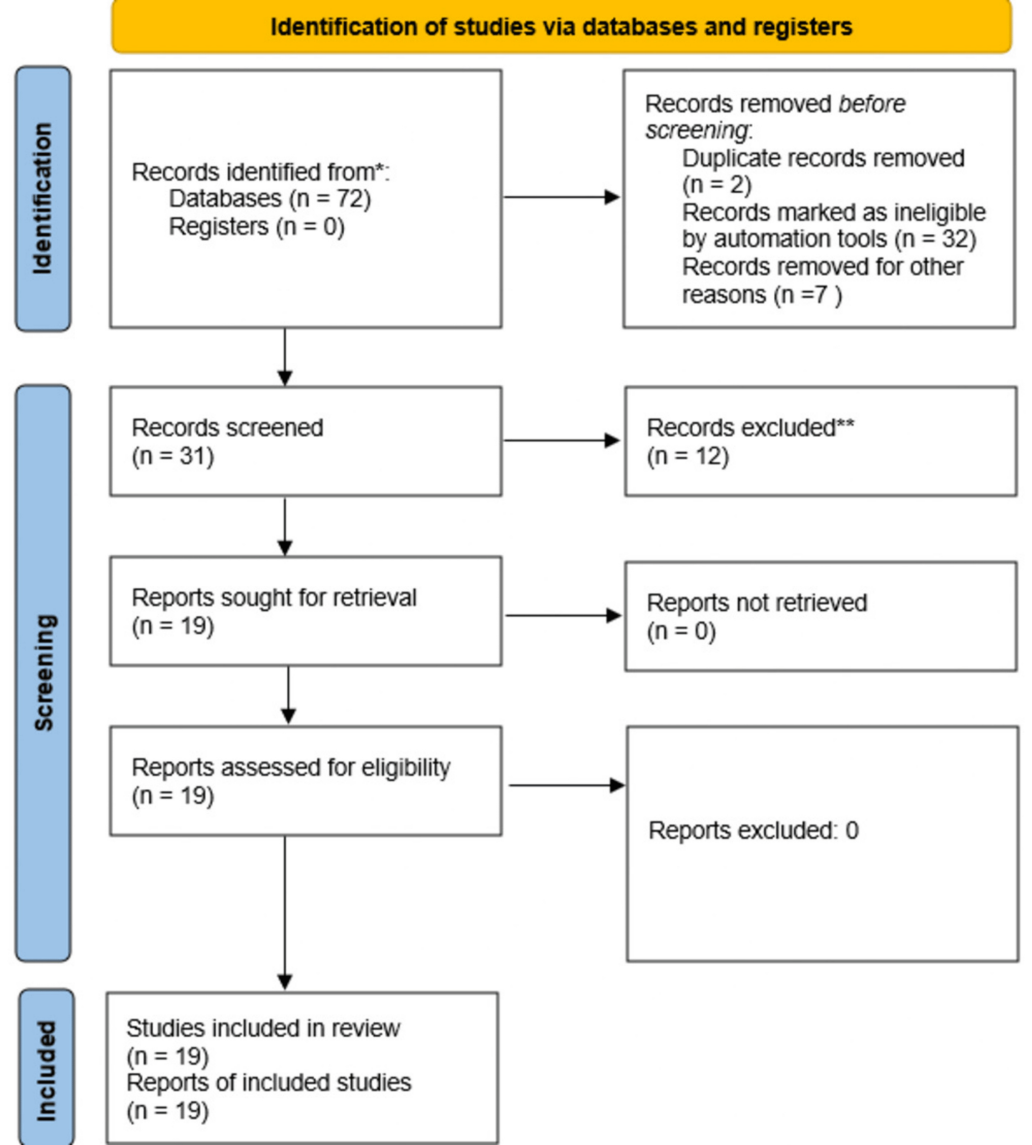
PRIMSA flow diagram for study screening and selection PRIMSA: Preferred Reporting Items for Systematic Reviews and Meta-Analyses

This systematic review was conducted in accordance with the Preferred Reporting Items for Systematic Reviews and Meta-Analyses (PRISMA) 2020 guidelines and was registered prospectively in PROSPERO (CRD420251112693). The review focused on the clinical presentation and histopathologic patterns of granulomatous skin reactions following tattooing or cosmetic micropigmentation. The PICO framework is presented in Table 1. A comprehensive electronic search was performed in PubMed and MEDLINE for English-language studies published between January 2000 and July 2025. The search strategy was compromised ("tattooing"[MeSH Terms] OR "tattoo*"[Title/Abstract] OR "permanent makeup"[Title/Abstract] OR "micropigmentation"[Title/Abstract]) AND ("granuloma"[MeSH Terms] OR "granulomatous reaction*"[Title/Abstract] OR "foreign body granuloma"[Title/Abstract] OR "sarcoidosis"[Title/Abstract]) AND ("clinical presentation"[Title/Abstract] OR "incidence"[Title/Abstract] OR "manifestation*"[Title/Abstract]). Only published studies were considered. No language or publication-status filters beyond English were applied. All identified records were imported into a screening spreadsheet and duplicated. Two reviewers independently performed a two-stage screening of titles and abstracts to exclude clearly irrelevant articles, followed by a full-text screening for eligibility based on predefined inclusion/exclusion criteria (Figure 1). Disagreements were resolved by discussion and consensus. Human studies describing granulomatous cutaneous reactions following tattooing or cosmetic micropigmentation were included, as were non-granulomatous inflammatory or infectious tattoo reactions. Animal studies, reviews, or editorials; duplicate or overlapping datasets without new extractable data; and articles without sufficient clinical or histological details were excluded.

**Table 1:** PICO framework

Component	Description
Population (P)	Individuals with tattoos or permanent cosmetic micropigmentation who developed cutaneous reactions
Intervention/exposure (I)	Exposure to tattoo pigments or tattooing procedures
Comparison (C)	Not applicable (no comparator group, as included studies were case reports and case series)
Outcome (O)	Granulomatous reactions at tattoo sites, tattoo-associated uveitis, development or unmasking of systemic sarcoidosis

**Figure 2:**
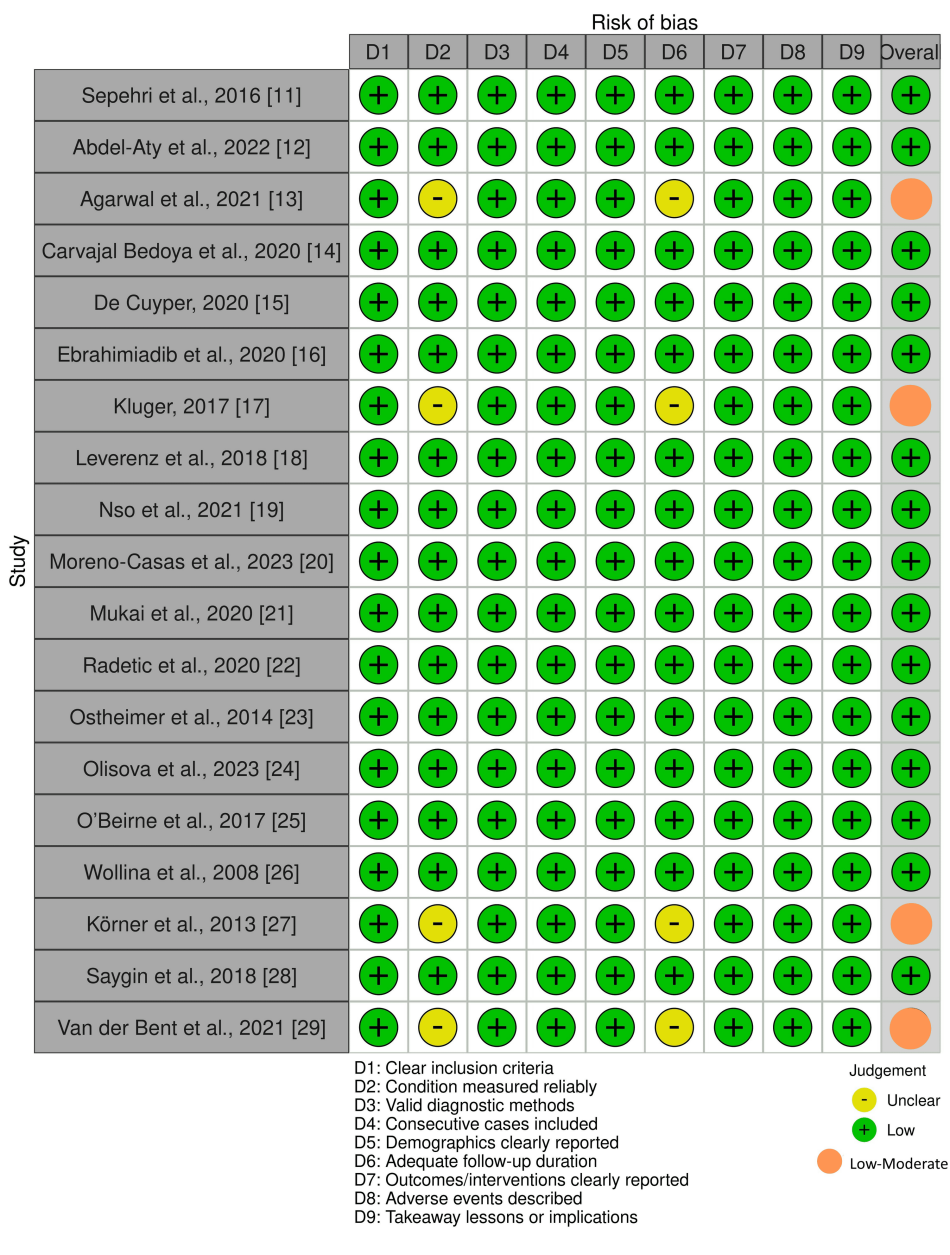
Risk of bias assessment using JBI critical appraisal tools JBI: Joanna Briggs Institute

Data extraction was performed using an Excel form (Microsoft Corp., Redmond, WA, USA). Where data were unclear or incomplete, efforts were made to interpret consistently across studies using textual or tabular cues. Methodological quality and risk of bias were assessed independently by two reviewers using the Joanna Briggs Institute (JBI) Critical Appraisal Tools appropriate to each study type [10]. Each item was scored as yes, no, unclear, or not applicable (Figure 2). Disagreements were resolved by consensus. The overall quality was summarized narratively as high, moderate, or low, depending on fulfillment of critical domains. Across the 19 included studies, methodological quality was generally high, with 15 (79%) rated as low risk and four (21%) as low-moderate risk of bias. Most reports clearly described demographics, diagnostic confirmation through biopsy or imaging, and follow-up outcomes. A narrative synthesis was performed following Synthesis Without Meta-analysis (SWiM) guidelines. Data were grouped by type of granulomatous reaction, type of procedure, tattoo pigment, clinical presentation, timing, treatment modality, and outcome.

A total of 72 records were identified through database searching. After removal of duplicate records (n = 2), automation-based exclusions (n = 32), and other pre-screening exclusions (n = 7), 31 records remained for screening. Following title and abstract screening, 12 records were excluded, and 19 full-text reports were assessed for eligibility. All 19 studies met the inclusion criteria and were included in the final qualitative synthesis (Table 2).

**Table 2:** Studies included in the systematic review

Author (year)	Country	Study design	Number of cases with tattoo-associated cutaneous granulomas	Type of tattoo	Associated systemic sarcoidosis
Sepehri et al. (2016) [11]	Denmark	Case series	44	Tattoo	Present in few cases
Abdel-Aty et al. (2022) [12]	United Kingdom	Case report	1	Tattoo	Present
Agarwal et al. (2021) [13]	India	Case series	5	Tattoo	Not assessed
Carvajal Bedoya et al. (2020) [14]	USA	Case report	1	Tattoo	Absent
De Cuyper et al. (2020) [15]	Belgium	Case report with review	1	Tattoo	Absent
Ebrahimiadib et al. (2020) [16]	Iran	Case series	2	Micropigmentation	Present
Kluger (2017) [17]	Finland	Case series	6	Tattoo	Present in some cases
Leverenz et al. (2018) [18]	USA	Case series	1	Tattoo	Present
Nso et al. (2021) [19]	USA	Case series	2	Tattoo	Present
Moreno-Casas et al. (2023) [20]	Spain	Case report	1	Tattoo	Absent
Mukai et al. (2020) [21]	Japan	Case report	1	Tattoo	Present
Radetic et al. (2020) [22]	USA	Case report	1	Tattoo	Absent
Ostheimer et al. (2014) [23]	USA	Case series	7	Tattoo	Present in some cases
Olisova et al. (2023) [24]	Russia	Case series	7	Micropigmentation	Present
O’Beirne et al. (2017) [25]	Ireland	Case report	1	Tattoo	Present
Wollina et al. (2008) [26]	Germany	Case report	1	Tattoo	Absent
Körner et al. (2013) [27]	Germany	Case series	5	Tattoo	Present in some cases
Saygin et al. (2018) [28]	USA	Case report	1	Tattoo	Present
Van der Bent et al. (2021) [29]	Netherlands	Case series	56	Tattoo and micropigmentation	Present in some cases

Nineteen studies met the inclusion criteria, comprising 10 case reports, five case series, one cross-sectional study, and three retrospective cohort studies. Across these studies, 144 patients with granulomatous reactions related to tattooing or micropigmentation were identified. Studies originated from 13 countries, with the highest representation from the USA (n = 6), followed by Germany, Belgium, the United Kingdom, and others, indicating broad geographical distribution [11-29]. The mean age across all patients was 34.9 years (range 17-58). Sex distribution was nearly equal, with 51 females and 43 males. Ethnicity data were variably reported. Caucasian and African American individuals were the most frequent, followed by Indian, Japanese, and Guyanese individuals. Most granulomatous reactions occurred following tattooing (n = 133), with 11 cases arising after micropigmentation. The predominant pigment associated with granuloma formation was black ink (n = 98), followed by red (n = 7), multicolour pigments (n = 2), red-black combinations (n = 3), and single isolated cases of dark blue, green, and violet ink. Four reports did not mention pigment color [12,16,18,28]. The most commonly involved sites included the arms (n = 45), followed by the torso (n = 14), face (n = 7), eyebrows (n = 5), lips (n = 2), back (n = 4), and lower limbs (n = 8). Thirteen patients presented with reactions in multiple or unspecified locations. The mean time from tattoo placement to symptom onset was 3.9 years, with a wide range of 0.16 to 20 years. The mean duration of symptoms before presentation was 9.4 months (range 0.03-50 months).

Granulomatous reactions manifested predominantly as papules and nodules (n = 55), swelling (n = 52), itch (n = 34), and induration (n = 8). Additional presentations included scaling, plaques, hyperpigmented lesions, linear bulges, pain (n = 10), and rare bullous reactions. These clinical patterns were consistent across both tattoo and micropigmentation procedures. Ocular involvement was documented in several studies, with uveitis reported in 20 patients and additional cases showing ocular inflammation or non-specific ocular findings. Histopathology consistently demonstrated non-caseating granulomas, reported in 97 patients, with an additional 41 patients showing sarcoidal granulomas. Other biopsy descriptions included non-necrotizing epithelioid granulomas, granulomas with retained pigment, and granulomas without visible pigment particles. Systemic sarcoidosis was documented in 41 patients across the included literature, highlighting the recognized association between tattoo granulomas and systemic granulomatous disease.

Treatment modalities were heterogeneous and tailored to disease severity. Oral corticosteroids were the most frequently used therapy (n = 10 as monotherapy; more when combined with other agents). Additional treatments included intralesional triamcinolone, topical corticosteroids, methotrexate, mycophenolate mofetil, tacrolimus (topical), allopurinol (topical), adalimumab, NSAIDs, and combination systemic immunosuppressants in select patients. The mean treatment duration was 5.07 months, with a range of 0.7 to 20 months. Of the 28 cases with clearly documented outcomes, 25 patients experienced partial or complete resolution, two had unclear outcomes, and one patient did not respond to therapy. Recurrence was reported in three cases, while seven cases showed no recurrence.

Discussion

This systematic review synthesizes evidence from 19 studies encompassing 144 patients with granulomatous cutaneous reactions following tattooing or cosmetic micropigmentation. The findings demonstrate that such reactions predominantly occur after tattooing rather than micropigmentation, with black ink being the most frequently implicated pigment. Clinical manifestations were heterogeneous but most commonly presented as papules, nodules, swelling, and pruritus, often with a delayed onset occurring several years after pigment deposition. Histopathological evaluation consistently revealed non-caseating or sarcoidal granulomas, frequently with retained pigment particles, underscoring a uniform granulomatous response pattern across diverse geographic settings. Notably, a substantial proportion of patients exhibited extracutaneous involvement, with systemic sarcoidosis documented in nearly one-third of cases and ocular involvement, particularly uveitis, reported in a notable subset. Treatment approaches were varied but largely immunosuppressive, most commonly involving corticosteroids, and outcomes were generally favorable, with the majority of reported cases achieving partial or complete resolution. However, variability in latency, presentation, and follow-up, along with the predominance of observational study designs, highlights the clinical heterogeneity and diagnostic challenges associated with tattoo-related granulomatous reactions.

Agarwal et al. [13] reported that among 1963 tattooed individuals, 1.1% developed tattoo reactions and 0.25% developed granulomas. Kluger et al. [17] noted that although 10-20% of Western adults have tattoos, only six of 31 tattoo complications in their cohort were granulomatous, and they highlighted topical corticosteroids as first-line therapy. Rebecca et al. [27] observed five granulomatous reactions among 19 tattoo complications. Sepehri et al. [11] identified eight cases of tattoo sarcoidosis among 72 papulonodular tattoo reactions, with systemic sarcoidosis present in 27 of these (5%) reactions (494) seen in their specialized clinic. Van der Bent et al. [29] documented 52 granulomas among 301 tattoo complications, of which 8 progressed to systemic sarcoidosis.

The predominance of sarcoid-like granulomatous reactions and the frequent association with systemic sarcoidosis observed in this review reinforce the concept that tattoo-related granulomas may represent more than a localized foreign-body response in a significant subset of patients. The delayed latency between pigment implantation and symptom onset, often extending over several years, suggests a complex immunological mechanism rather than an acute inflammatory reaction. Black ink emerged as the most commonly implicated pigment, which may reflect its widespread use rather than its intrinsic antigenicity; however, its frequent association across studies raises the possibility that specific pigment constituents or degradation products act as chronic immune triggers. The wide variability in clinical presentation and timing, combined with the consistent histopathological findings, highlights the diagnostic challenge posed by these lesions, which can mimic isolated cutaneous sarcoidosis, foreign-body granulomas, or other inflammatory dermatoses. Notably, the occurrence of ocular and systemic involvement underscores the need for clinicians to view tattoo-associated granulomatous reactions as potential markers of underlying systemic disease rather than purely localized complications.

The biological plausibility of granulomatous reactions following tattooing or cosmetic micropigmentation is supported by established mechanisms of chronic antigen-driven immune activation in the skin. Tattoo pigments are permanently deposited in the dermis, where macrophages phagocytose them and can persist intracellularly for years, providing a sustained antigenic stimulus that favors granuloma formation [1]. Non-caseating granulomas represent a T-helper-1-mediated immune response characterized by macrophage activation and epithelioid cell transformation, a pathway shared by both foreign-body granulomas and sarcoidosis [30,31]. Experimental and clinicopathologic studies have demonstrated that certain tattoo pigments, particularly black inks containing carbon-based particles, polycyclic aromatic hydrocarbons, and metal contaminants, may undergo slow degradation or migration, further amplifying immune recognition over time [32,33]. This mechanism is consistent with the prolonged latency observed between tattoo placement and clinical presentation in the included studies. Additionally, tattoo-associated granulomas have been proposed to act as sites of immune unmasking in genetically predisposed individuals, potentially triggering systemic sarcoidosis rather than merely reflecting localized cutaneous disease [34,35]. The documented association with ocular and systemic involvement supports this hypothesis and suggests that tattoo granulomas may serve as clinically visible indicators of an underlying systemic granulomatous tendency [36].

The included evidence is predominantly derived from case reports and small case series, with only a limited number of observational studies, which restricts the ability to draw causal inferences and precludes quantitative meta-analysis. Considerable heterogeneity existed across studies in clinical presentation, latency period, pigment color reporting, histopathological terminology, systemic evaluation for sarcoidosis, treatment strategies, and follow-up duration, limiting meaningful comparisons and synthesis. The findings of this review have important clinical implications, particularly for dermatologists, pathologists, and plastic surgeons involved in aesthetic procedures. Granulomatous reactions occurring within tattoos or cosmetic micropigmentation should prompt thorough clinical evaluation, including histopathological confirmation and assessment for systemic involvement, especially sarcoidosis and ocular disease. Awareness of the potential for delayed presentation underscores the need for long-term vigilance rather than reassurance based solely on the tattoo's age. From a public health and regulatory perspective, the frequent association with specific pigment colors underscores the importance of greater transparency into tattoo ink composition and standardized safety regulations. Future research should prioritize prospective, multicenter studies with standardized reporting of pigment constituents, latency periods, diagnostic work-up, and long-term outcomes. Such efforts would facilitate earlier diagnosis, improve risk stratification, and guide evidence-based management strategies for affected individuals.

## Conclusions

Granulomatous reactions following tattooing and cosmetic micropigmentation are delayed, immune-mediated complications that may signify underlying systemic sarcoidosis rather than isolated cutaneous disease. These reactions most commonly involve black ink, can present years after pigment deposition, and frequently demonstrate non-caseating granulomas on histopathology. Given the notable association with ocular and systemic involvement, clinicians should pursue biopsy confirmation and appropriate systemic evaluation. Although immunosuppressive therapy is generally effective, heterogeneity in presentation and follow-up highlights the need for standardized diagnostic pathways and prospective studies to guide risk stratification and management.
